# Is jugular bulb oximetry monitoring associated with outcome in out of hospital cardiac arrest patients?

**DOI:** 10.1007/s10877-020-00530-x

**Published:** 2020-05-20

**Authors:** Jaromir Richter, Peter Sklienka, Adarsh Eshappa Setra, Roman Zahorec, Samaresh Das, Nilay Chatterjee

**Affiliations:** 1 Department of Anesthesiology and Intensive Care Medicine, University Hospital Ostrava, Ostrava, Czech Republic; 2 Department of Anaesthetics, Yeovil Hospital, Greater Kingston, Somerset, BA21 4AT UK; 3 Second Department of Anesthesiology and Intensive Medicine, Medical School, Comenius University, Bratislava, Slovak Republic

**Keywords:** Brain hypoxia, Jugular bulb oxygen saturation, Out of hospital cardiac arrest, Cerebral edema, Monitoring

## Abstract

Cerebral protection against secondary hypoxic-ischemic brain injury is a key priority area in post-resuscitation intensive care management in survivors of cardiac arrest. Nevertheless, the current understanding of the incidence, diagnosis and its’ impact on neurological outcome remains undetermined. The aim of this study was to evaluate jugular bulb oximetry as a potential monitoring modality to detect the incidences of desaturation episodes during post-cardiac arrest intensive care management and to evaluate their subsequent impact on neurological outcome. We conducted a prospective, observational study in unconscious adult patients admitted to the intensive care unit who had successful resuscitation following out of hospital cardiac arrest of presumed cardiac causes. All the patients were treated as per European Resuscitation Council 2015 guidelines and they received jugular bulb catheter. Jugular bulb oximetry measurements were performed at six hourly intervals. The neurological outcomes were evaluated on 90th day after the cardiac arrest by cerebral performance categories scale. Forty patients met the eligibility criteria. Measurements of jugular venous oxygen saturation were performed for 438 times. Altogether, we found 2 incidences of jugular bulb oxygen saturation less than 50% (2/438; 0.46%), and 4 incidences when it was less than 55% (4/438; 0.91%). The study detected an association between SjVO_2_ and CO_2_ (r = 0.26), each 1 kPa increase in CO_2_ led to an increase in SjvO_2_ by 3.4% + / − 0.67 (p < 0.0001). There was no association between SjvO_2_ and PaO_2_ or SjvO_2_ and MAP. We observed a statistically significant higher mean SjvO_2_ (8.82% + / − 2.05, p < 0.0001) in unfavorable outcome group. The episodes of brain hypoxia detected by jugular bulb oxygen saturation were rare during post-resuscitation intensive care management in out of hospital cardiac arrest patients. Therefore, this modality of monitoring may not yield any additional information towards prevention of secondary hypoxic ischemic brain injury in post cardiac arrest survivors. Other factors contributing towards high jugular venous saturation needs to be considered.

## Introduction

Annually, about 1 in 1000 people experience out of hospital cardiac arrest (OHCA), of which about 25% survive to hospital admission and only 10% survive until hospital discharge [1]. The most common cause of death in OHCA patients who survive to intensive care unit (ICU) admission is severe neurological injury which is a summation of primary and secondary hypoxic ischemic brain injuries, HIBI [2, 3]. The primary HIBI is caused by cessation of cerebral blood flow during the cardiac arrest and it resumes with the return of spontaneous circulation (ROSC). Subsequently there is a period characterized by an imbalance between cerebral oxygen demand and cerebral oxygen delivery which may occur within minutes, hours or days after ROSC. This results in secondary HIBI. The cerebral protection strategies against secondary HIBI remains the key priority of post-cardiac arrest (post-CA) ICU management.

There is a paucity of information regarding the incidence of cerebral hypoxia following OHCA in medical literature. Nevertheless, no ideal method for bedside monitoring of cerebral oxygenation has been identified. Commonly employed methods for cerebral oxygenation are: (i) jugular bulb oxygen saturation (SjvO_2_), (ii) brain tissue oxygen tension (PbtO_2_), and (iii) near-infrared spectroscopy, NIRS [4]. SjvO_2_ monitoring is a relatively simple method, which measures the oxygen saturation of the blood returning from cerebral circulation via a catheter inserted retrograde into the internal jugular vein and advanced into the jugular bulb. It can be continuous or intermittent process which reflects the global balance between cerebral oxygen supply and consumption according to the Fick principle. SjvO_2_ has been used extensively in patients with severe traumatic brain injury (TBI). Brain Trauma Foundation (BTF) 2016 guidelines for management of severe TBI mentions only SjvO_2_ (level III recommendations) as advanced cerebral monitoring for detecting cerebral hypoxia (SjvO_2_ < 50% is considered as threshold) [5]. Brain tissue oxygen tension monitoring (PbtO_2_) involves direct estimation of regional brain tissue oxygen tension by introducing a small catheter into the brain parenchyma. The prime disadvantage of this method is its invasiveness. NIRS has been considered to be a promising method for non-invasively assessing cerebral oxygenation. The changes in cerebral oxygenation derived from NIRS might reflect the changes in relationship between oxygen delivery and consumption in the brain [6]. However, some of the recent studies conducted in brain-dead patients demonstrated that NIRS is more likely to be affected by extracranial perfusion status rather than truly reflecting intracranial perfusion [7, 8]. Because of this presently NIRS may not be considered to be an ideal routine monitoring device for cerebral oxygenation [9].

The aims of the present study were (i) to evaluate SjvO_2_ as a possible monitoring modality for detecting desaturation episodes during post-CA ICU care and (ii) assessing the impact of secondary HIBI (arising during desaturation episodes of SjvO_2_) on neurological outcome of the patients.

## Materials and methods

### Study design

This prospective, observational, single centre study was performed from July 2016 to September 2018 in a tertiary care ICU (admitting mixed medical/surgical patient population) in University Hospital Ostrava, Czech Republic. The clinicians were blinded to the SjvO_2_ results. The study protocol and informed consent procedures were approved by Local Medical Ethics Committee (Ethics Committee of FN Ostrava Ref No. 410/2016). Because of patients’ clinical state it was not feasible to obtain informed consent at the time of intervention. Therefore, a deferred informed consent from the patient’s next of kin was obtained as soon as possible. In addition, we obtained informed consent from all the patients who regained sufficient neurological function (Cerebral Performance Category CPC 1–2). The study was registered with ClinicalTrials.gov (Identifier NCT02806778).

### Patients

We consecutively screened all unconscious adult patients who were admitted to the ICU following OHCA between July 2016 and September 2018. The inclusion criteria were (i) age > 18 years, (ii) all patients who had OHCA of presumable cardiac cause, (iii) Glasgow Coma Scale (GCS) </= 8 following ROSC and unable to follow verbal commands. The exclusion criteria were (i) pregnancy, (ii) OHCA secondary to any non-cardiac cause, (iii) severe comorbid conditions resulting in limited life expectancy, (iv) confirmed acute intracranial bleeding, (v) greater than 6 h (360 min) of duration between the time of cardiac arrest and the time of first jugular bulb blood sampling, and (vi) patients who died within 72 h from cardiac arrest due to refractory post-CA shock.

### Management protocol

All the patients admitted to the study received out-of-hospital advanced life support performed by an emergency team, which included a physician trained in emergency medicine. Patients who had successful ROSC were transported to the Emergency Department at our Tertiary Care Centre. Patients with a presumed cardiac origin of OHCA were immediately taken to the catheterization laboratory for a coronary angiogram (followed by a percutaneous coronary intervention, if indicated) according to the decision made by a consultant cardiologist. When the cardiac cause of OHCA was not confirmed, a head and chest computed tomography (CT) scan were performed to evaluate neurological or respiratory etiology.

Thereafter, patients were admitted to the ICU for post-resuscitation care according to European Resuscitation Council (ERC) guidelines (SaO_2_ 94–98%, PaCO_2_ 4.6–6.0 kPa; optimal mean arterial pressure (MAP) to achieve adequate urine output and normal or decreasing trend of plasma lactates, adequate sedation and early diagnosis/treatment of seizure) [10]. Following the institutional protocol the core temperature of all the patients were maintained between 35 and 36 °C for 24 h using external surface cooling technique. Patients were sedated with sufentanyl, propofol and/or midazolam. The aim of sedation during the targeted temperature management (TTM) was to achieve Richmond agitation sedation score (RASS) within the range of − 4 to − 5 and to eliminate clinical seizures and shivering [11]. The arterial and central venous catheters were inserted in all the patients. In addition, a single lumen catheter was inserted retrograde into the ultrasonographically determined dominant jugular vein or in the right jugular vein [12]. The final position of the tip of the catheter was confirmed above the lower border of the C1 vertebra, as confirmed on a lateral cervical spine X-ray [13]. Blood samples were drawn gently through the jugular vein catheter for SjvO_2_ sampling to prevent any retrograde admixture.

### Data collection and interpretation

The first blood sample for SjvO_2_ was drawn from the jugular bulb catheter immediately following ICU admission and jugular bulb cannulation (time T_0_). Then serial samples were taken at every 6 h until 72 h post-CA (time T_66_) or until the time of extubation. According to the study protocol, jugular bulb blood samples were blinded to the laboratory and the treating physician was unaware about the SjvO_2_ results. Thus, no diagnostic or therapeutic interventions were performed based on SjvO_2_ values. Arterial blood samples were taken simultaneously with jugular bulb samples, and MAP values were recorded at each of these points of time.

### Outcome assessment

The primary outcome was the incidence of secondary HIBI, as detected by the jugular bulb oximetry. Brain hypoxia was considered as SjvO_2_ < 50% according to BTF 2016 guidelines [5]. The secondary outcome was to determine any association between the incidences of secondary HIBI and the neurological outcomes in this population. Patients were evaluated by a consultant neurologist 90 days after their cardiac arrest. CPC 1 and 2 were considered as a favorable outcome, whereas CPC 3, 4 and 5 were considered as a unfavorable outcome [14].

### Statistics

The data obtained from the study was expressed in the tabular form (Tables 1, 2, 3). The normality of the numeric variables was tested using the Shapiro–Wilk test and a logarithmic transformation was applied in order to normalize the skewed data when required. Numerical data were expressed as mean +/− standard deviation (SD) or median and 25–75% inter-quantile range. The numerical variables between the groups were compared using paired t-test or Wilcoxon rank sum test. The categorical variables were compared with chi square test or Fisher’s exact test, as applicable. A linear mixed model was used to demonstrate the correlations between SjvO_2_ and MAP, PaCO_2_, PaO_2_. A p value < 0.05 was considered to be statistically significant. R statistical software version 3.5.2 (R Core Team 2019) was used for the analysis.

**Table 1:** Study variables

Variables	Favorable outcome	Unfavorable outcome	Total	p
Number of patients	17	23	40	
Gender (% of male)	15/17 (88)	21/23 (91)	36/40 (90)	NS
Age (years) (mean + / − SD)	55.47 + / − 10.84	61.39 + / − 9.66	58.88 + / − 10.47	NS
Time of arrest to ROSC (min) (mean + / − IQR)	14/17 (8–25)	21/23 (18–33)	20.5 (14–29.3)	0.012
Initial rhythm				
Asystole/PEA (%)	0/17 (0)	7/23 (30)	7/40 (18)	0.014
Shockable rhythm (%)	17/17 (100)	16/23 (70)	33/40 (82)	0.014
APACHE II (mean + / − IQR)	25 (23.5–27)	29 (26.5–33)	28 (25–30)	0.014
Initial tests				
Hemoglobin (g/dl) (mean + / − SD)	14.4 + / − 1.7	14.1 + / − 1.6	14.2 + / − 1.6	NS
pH (mean + / − SD)	7.27 (7.25–7.33)	7.27 (7.11–7.37)	7.27 (7.18–7.36)	NS
Lactate (mmol/l) (mean + / − IQR)	1.9 (1.3–3.9)	2.2 (1.65–6.15)	2.1 (1.3–5.72)	NS
Underwent coronary angiography (%)	16/17 (94)	22/23 (96)	38/40 (95)	NS
Underwent PCI + coronary angiography (%)	10/17 (59)	13/23 (57)	23/40 (58)	NS

*SD* standard deviation, *IQR* 25–75% inter quartile range

**Table 2:** SjvO_2_ levels at different time intervals and outcomes

SjvO_2_ values	Favorable outcome (mean + /** − **IQR)	Unfavorable outcome (mean + /** − **IQR)	p
T_0_	75 (68–79)	75 (63.5–78.5)	NS
T_6_	77 (72–80)	79 (69.5–82)	NS
T_12_	75 (72–83)	81 (77–86)	NS
T_18_	74.5 (68.5–78.25)	83 (79–87.5)	0.0003
T_24_	76 (71–80)	84 (78–87.5)	0.004
T_30_	78 (71–81)	83 (77–85.5)	0.01
T_36_	75 (62.5–79)	82 (76–86.5)	0.007
T_42_	71 (64.25–78.75)	81 (78.25–84.75)	0.0006
T_48_	77 (66–78)	82.5 (80–85)	0.002
T_54_	70 (68–77)	82 (81–86)	< 0.0001
T_60_	72 (67.5–76)	83 (79.25–86.5)	0.003
T_66_	71 (67.25–74.25)	81.5 (79.25–85.75)	0.0004

**Table 3:** Incidence of SjvO_2_ < 50% and < 55% and relation with outcomes

SjvO_2_	Favorable outcome (n = 17)	Unfavorable outcome (n = 23)	Overall (n = 40)
SjvO_2_ (no of measurements)	170 (39%)	268 (61%)	438 (100%)
SjvO_2_ < 50% (number of episodes)	1	1	2 (0.46%)
SjvO_2_ < 55% (number of episodes)	3	1	4 (0.91%)

## Results

From July 2016 until September 2018, 54 consecutive OHCA patients of cardiac origin were presented to our Tertiary Care Center. Fourteen of them were excluded, as they did not meet the inclusion criteria (8 died within 72 h due to refractory post CA shock, 3 were found to have a non-cardiac cause of CA, 1 had intracranial bleeding after sustaining a fall following OHCA, in 1 patient jugular bulb catheter could not be inserted, 1 had accidental removal of the jugular bulb catheter during X ray).

Therefore, 40 patients were included in the final analysis. The demographic profile of the patients and the study variables are presented in Table 1. 17 Patients (43%) survived with a CPC 1 or 2 on day 90 post-CA. The median duration from the time of the cardiac arrest to the time of taking the first blood sample from the jugular bulb catheter was 250 min.

SjvO_2_ values were measured at every 6 h (T_0_ to T_66_ or until extubated) (Table 2). Altogether SjvO_2_ measurements were performed 438 times (Table 3). SjvO_2_ values of less than 50% were detected twice (2/438; 0.46%) in 2 different patients (1 had favorable and the other had unfavorable outcome); whereas SjvO_2_ values of less than 55% were detected 4 times (4/438; 0.91%) in 3 different patients (2 had favorable and 1 had unfavorable outcome) (Table 3).

More frequent incidence of low SjvO_2_ was detected in the group of patients who died within 72 h of ICU admission due to post-CA shock and were excluded from the statistics. This subgroup consisted of 8 patients out of them 7 died within the first 24 h from cardiac arrest. The mean from the time of ICU admission to death was 11 h. Six of them had two SjvO_2_ samples drawn and 1 patient had only 1 SjvO_2_ sample. SjvO_2_ < 50% was detected three times (3/13, 23%) and each time it was found in the last drawn sample, taken before they died.

**Fig. 1:**
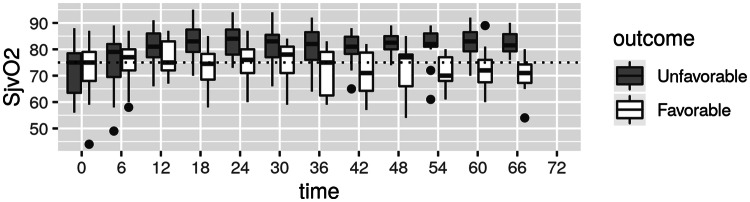
SjvO_2_ levels at different time intervals in favorable and unfavorable groups

**Fig. 2:**
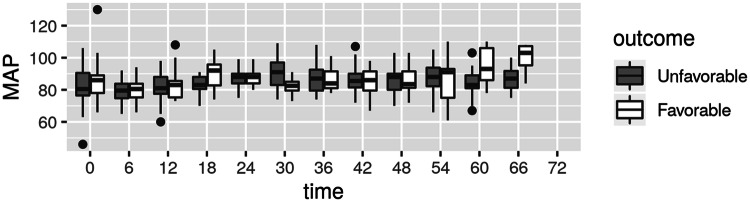
MAP levels at different time intervals in favorable and unfavorable groups

Significantly higher mean SjvO_2_ values were detected in unfavorable outcome group from time T_18_ until the end of measurement time (Table 2; Fig. 1). No significant difference was found in PaCO_2_ values between the groups with favorable or unfavorable outcomes. MAP was comparable between the groups at all time points except at T_18_ (mean 92; range 82.7 to 95.7 and mean 83; range 80.5 to 88 in favorable and unfavorable groups respectively, p = 0.028) and at T_30_ (mean 82.5; range 79.5 to 85 and mean 91; range 83 to 97 in favorable and unfavorable groups respectively, p = 0.025) (Fig. 2).

**Fig. 3:**
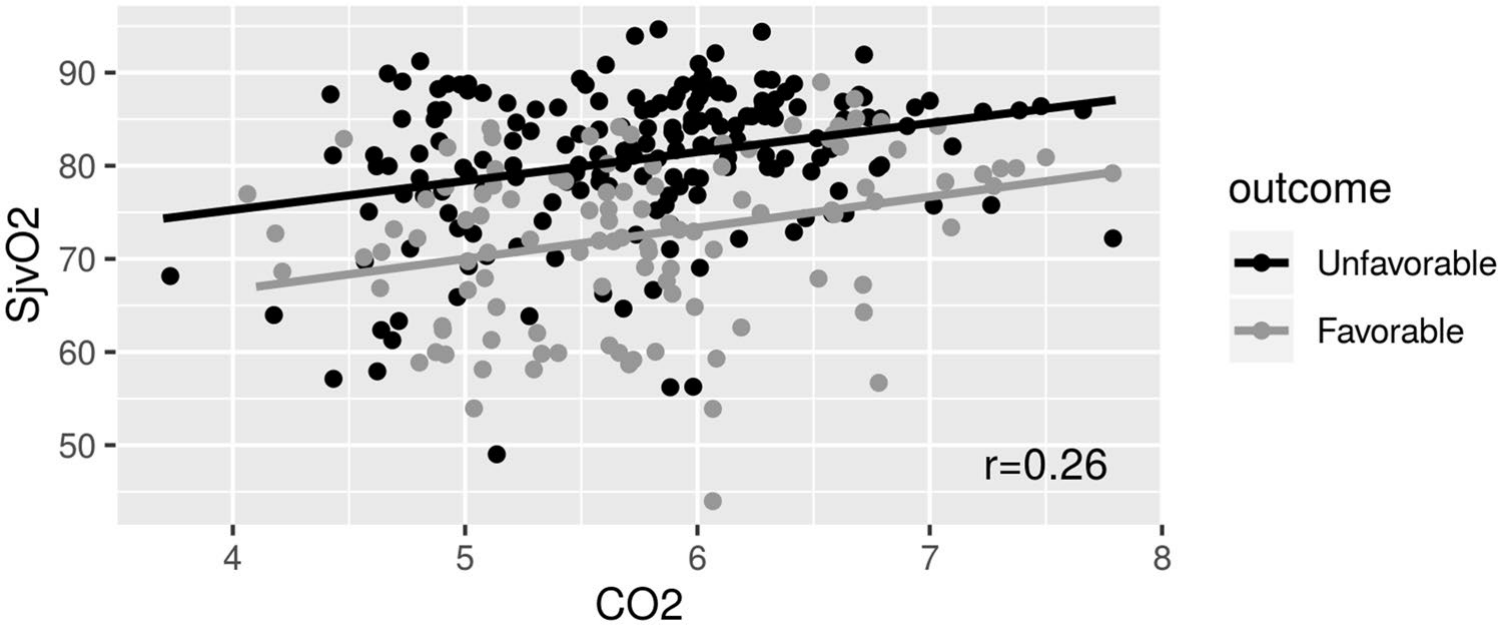
Correlation between SjvO_2_ and PaCO_2_

A linear mixed model used to assess the correlation of SjvO_2_ with CO_2_, MAP and PaO_2_. The unfavorable outcome group was having a statistically significant higher mean SjvO_2_ (8.82% +/− 2.05, p < 0.0001) compared to the favorable group. An association was found between SjvO_2_ and CO_2_ (r = 0.26), each 1 kPa increase in CO_2_ led to an increase in SjvO_2_ by 3.4% +/− 0.67 (p < 0.0001) (Fig. 3). No similar associations were found between SjvO_2_ and PaO_2_ (r = 0.19) or SjvO_2_ and MAP (r = 0.02).

The second aim (impact on neurological outcome) could not be assessed due to very low incidence of desaturation episodes.

## Discussion

Secondary HIBI is considered to be an important factor for adverse outcomes in patients suffering from CA, but there is paucity of information about the precise incidences of brain hypoxic events and which modality is best to detect it. The present study describes the incidences of cerebral hypoxia as detected by jugular bulb oximetry in post-OHCA population. Most of the existing clinical experiences concerning this method is in patients with severe TBI. Previous studies demonstrated associations between secondary brain hypoxia detected by jugular bulb oximetry during ICU care and mortality in victims of TBI [15, 16]. BTF recommends jugular bulb monitoring of arteriovenous oxygen content difference, as a source of information for management decisions in severe TBI patients (advanced cerebral monitoring, level III recommendations); with a threshold of SjvO_2_ of less than 50% in order to reduce mortality and improve outcome [5]. Because of the paucity of guidelines and thresholds of SjvO_2_ in post-CA patients we extrapolated cut off point of SjvO_2_ from TBI [5].

The most significant finding from the study was an extremely low incidence of desaturation episodes (SjvO_2_ < 50% in 0.46% of the patients). Even considering a more conservative SjvO_2_ threshold (i.e. SjvO_2_ < 55%) following an extensive literature search [17, 18], could not yield any remarkable difference on the findings (0.91%).

Wallin et al. had conducted a prospective study over in 75 adult post-CA comatose patients who had successful ROSC. 49% of their patients had survived with good neurological outcome at 6 months post-CA, but there was no significant difference in SjvO_2_ between the patients of good and poor outcome [19]. However, their study included a case mix of OHCA and in hospital CA patients recruited between 2004 and 2012, before the publication of ERC 2015 guidelines. In a recent study with 10 patients of post-CA with a risk of brain injury by Sekhon and colleagues; none of the patients had SjvO_2_ less than 50% but 38% of the times they experienced a brain tissue oxygen below 20% (by simultaneously monitoring PbtO_2_) [20]. Another interesting results from the study was the association between SjvO_2_ and CO_2_ (r = 0.26) (Fig. 3), but no similar associations were found between SjvO_2_ and PaO_2_ (r = 0.19) or SjvO_2_ and MAP (r = 0.02).

There could be several explanations for the low incidences of cerebral desaturation episodes. Post-resuscitation ICU care has greatly improved in recent times. Some studies suggested that by implementing strict therapeutic protocols focused on optimum hemodynamics and TTM in post-CA care resulted in improved survival [21, 22]. Implementation of deep sedation regimen could be another potential reason. The existing guidelines recommend for adequate sedation during TTM. There could be considerable variation in sedation protocols amongst individual ICUs [23]. Deep sedations can reduce cerebral oxygen consumption up to 60% compared to baseline in a dose dependent manner [24–27]. It also suppresses convulsive/nonconvulsive seizures and prevent shivering [10, 28]. Sedation was aimed for a RASS between − 4 and − 5 in the present study, however only RASS of − 5 was recorded at all times during TTM. There were no episodes of convulsive seizures or shivering in our cohort. Deep sedation might have a potential for cerebral protection [29].

Increased diffusion barriers secondary to cerebral edema may play an important role. Cerebral edema can occur following CA and its extent as evidenced by CT or magnetic resonance imaging (MRI) may be used as prognosticator of neurological outcome. It can manifest as early as 1 h following successful resuscitation [30] and subsequently worsens in patients with unfavorable outcome [31, 32]. It is probable that hemodynamic optimization provides adequate oxygen delivery to the cerebral microcirculation, but formation of cerebral edema may reduce oxygen diffusion out of the microcirculation, thus SjvO_2_ can remain ‘arterialized’ [33]. This could be the reason for discrepancies between SjvO_2_ and PbtO_2_ measurements in the study by Sekhon et al. [20]. The present study findings also support this hypothesis. Patients in both the groups (favorable and unfavorable) initially had similar SjvO_2_ values, but at time T_18_ onwards SjvO_2_ values were significantly higher in patients with unfavorable neurological outcome. Other studies have also shown similar trends in SjvO_2_ [34, 35]. Therefore, in future, it seems logical to consider the means of reducing cerebral edema as the principal therapeutic goal for neuroprotection and subsequently a better neurological outcome in post-CA patients [36].

The initial systemic lactate levels were remarkably low in our cohort. That might have been due to the fact that samples were not taken immediately on arrival to the hospital but collected only after ICU admission. By this time majority of the patients had percutaneous coronary intervention which might have resulted in better systemic perfusion. Moreover, patients with refractory post-CA circulatory failure were expected to have higher mean lactate levels, died within first 72 h. Hence they were excluded from the statistical analysis according to the study protocol.

Our study has few important limitations. Firstly, it is a single centre study with relatively small number of patients. Secondly, jugular bulb oximetry was performed intermittently and frequency of SjvO_2_ sampling was low. Therefore, there is a theoretical probability that some of secondary hypoxic episodes could have been missed. The possibility of existence of regional brain ischemia could not be excluded as SjvO_2_ reflects global cerebral oxygenation. Regional cerebral ischemia (focal) could occur in spite of normal SjvO_2_ values [37]. Finally, this study did not show significant number of episodes brain hypoxia exclusively using SjvO_2_ as a monitoring modality, the lack of simultaneous use of other intracranial monitoring (e.g. ICP and/or PbtO_2_) is a limitation.

The present study could detect very limited incidences of cerebral desaturation episodes using jugular bulb oximetry. The cause for this could me multifactorial. A statistical correlation with the neurological outcome cannot be computed as the incidences of low SjvO_2_ were rather rare. The findings from the present study can be further validated by a prospective multicentre study involving larger number of patients with adequate statistical power and using continuous jugular oximetry monitoring. Such study could also potentially include intracranial monitoring thus obtaining correlations between intracranial and SjvO_2_ values. Further research on this subject should probably focus on post-resuscitation brain edema as one of the potential therapeutic targets.
